# Finding a niche for cardiac precursors

**DOI:** 10.7554/eLife.02993

**Published:** 2014-05-06

**Authors:** Benoit G Bruneau

**Affiliations:** 1 **Benoit G Bruneau** is in the Gladstone Institute of Cardiovascular Disease, and Roddenberry Center for Stem Cell Biology and Medicine at Gladstone, San Francisco, United States, and the Department of Pediatrics and Cardiovascular Research Institute, University of California, San Francisco, United Statesbbruneau@gladstone.ucsf.edu

**Keywords:** cardiac progenitor, self-renewal, niche, numb, microenvironment, heart, mouse

## Abstract

Within an embryo, a region next to the developing heart provides a niche where cardiac precursor cells can increase in number before they contribute to the development of this organ.

**Related research article** Shenje LT, Andersen P, Uosaki H, Fernandez L, Rainer PP, Cho G-s, Lee D-i, Zhong W, Harvey RP, Kass DA, Kwon C. 2014. Precardiac deletion of Numb and Numblike reveals renewal of cardiac progenitors. *eLife* **3**:e02164. doi: 10.7554/eLife.02164

**Image** Chimeric mouse embryo with wild-type cells (green) and mutant cardiac precursors (red)

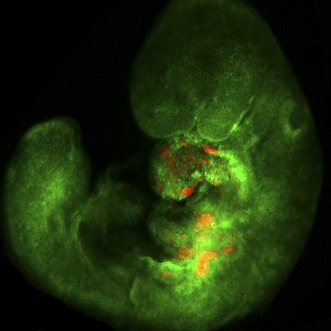

The heart is the first organ to form during the development of an embryo, and it starts to pump blood while it is still forming. Heart development requires ‘cardiac progenitor cells’ to be in precisely the right place within the embryo, and to then undergo changes to form the different tissues and structures that make up a heart (Evans et al., 2010; Bruneau, 2013). Although populations of cardiac progenitor cells have been identified in embryos, it is not known if these cells can replace themselves during development, or where within the embryo a self-renewing population of cells might thrive.

Progenitor cells are similar to embryonic stem cells. However, whereas stem cells can replicate indefinitely and are able to become a wide variety of specialized cell types, progenitor cells typically undergo only a limited number of replications and rapidly change into a limited number of cell types. For both stem cells and progenitor cells—which can be collectively called precursor cells—a ‘niche’ is somewhere within the embryo that encourages the cells to replicate without changing into more specific cell types (Scadden, 2014). Being able to ‘trap’ cardiac precursors in an environment that recreates such a niche would be an important first step for researchers hoping to develop regenerative techniques to repair damaged heart tissue.

Cardiac precursors can be found in embryonic regions that are immediately adjacent to the forming heart, in populations that are referred to as the ‘first’ and ‘second heart fields’ (which are named according to the order in which they appear in the embryo). These two precursor populations contribute to different parts of the heart; the first heart field forms the atria and left ventricle, whereas the rest of the heart is derived from the second heart field (Evans et al., 2010; Bruneau, 2013). Now, in *eLife*, Chulan Kwon of the Johns Hopkins University and co-workers—including Lincoln Shenje and Peter Andersen as joint first authors—report that they have identified a niche containing cardiac precursors that are maintained in a progenitor state by two proteins called Numb and Numblike (Shenje et al., 2014).

Shenje, Andersen et al.—who are based at Johns Hopkins, Yale University and the Victor Chang Cardiac Research Institute—discovered that deleting the genes for both Numb and Numblike causes severe defects in mice. Parts of the heart that are derived from the second heart field (namely the outflow tract and right ventricle) failed to develop normally. Furthermore, the second pharyngeal arch (PA2 for short)—an embryonic structure that is next to the top of the developing heart—was also smaller. Shenje, Andersen et al. determined that, shortly after the heart starts to develop, cardiac progenitor cells that would normally contribute to the outflow tract, right ventricle and PA2 were missing in these mutant mice. In wild-type mice, on the other hand, cells in the PA2 continued to proliferate as progenitor cells while they remained in this arch, and contributed directly to the heart by migrating into it as it developed. This suggests that the progenitor cells within the PA2 are a self-renewing population of cardiac precursors.

To examine the PA2 cells further, Shenje, Andersen et al. isolated cells from mouse embryos, and grew them in the lab on a substrate that mimics the extracellular matrix that surrounds cells in tissues. These ‘explant cultures’ revealed that, when released from their embryonic environment, some PA2 cells migrate out and spontaneously change into cardiomyocytes (heart muscle cells that can ‘beat’). Further, transferring cardiac precursors purified from embryonic stem cells onto a layer of PA2 cells maintained them in a self-renewing state and thus prevented them from changing into specific cell types. However, once removed from the co-culture with the PA2 cells, the cardiac precursor cells were released from their stem cell-like state and continued to differentiate into cardiomyocytes. The fact that the PA2 cells can maintain cardiac precursors, at least in culture, in a self-renewing progenitor state is striking because cardiac precursors taken from embryonic stem cells will never normally pause their differentiation into cardiomyocytes (Kattman et al., 2006).

Having defined the PA2 as a potential niche for cardiac precursors, Shenje, Andersen et al. set out to determine the role of Numb and Numblike in this embryonic region. These two proteins are known to maintain a progenitor state in the precursors of nerve cells (Petersen et al., 2002). Shenje, Andersen et al. used chimeric mice generated by taking stem cells from embryos with the *Numb/Numblike* genes knocked out (and also labeled with a red fluorescent protein), and injecting these cells into wild-type embryos. The combined loss of *Numb* and *Numblike* did not affect the migration of cardiac precursors into PA2, but these double knockout cells failed to proliferate when they reached the PA2. Thus, the Numb and Numblike proteins function within individual cells to regulate the multiplication of cardiac precursors with the PA2 niche. These findings provide a redefined view of progenitor allocation in heart development, and highlight the importance of a population of self-renewing cells within a niche.

Several important questions remain. Firstly, what is the nature of the signal within the PA2 that maintains cardiac precursors in a self-renewing state? Cardiac precursors derived from stem cells tend to rapidly differentiate into their final cell type, so finding this signal within the PA2 might allow the production of large numbers of cells for transplantation or in vitro organ generation (Laflamme and Murry, 2011). Harnessing the properties of the PA2 that promote self-renewal of cardiac precursors would allow this.

Another question is: does a similar niche exist for the other half of the heart (the atria and left ventricle)? If the answer to this question is no it would imply significant differences in the ways that precursor cells from these two heart fields contribute to the development of the heart.

Finally, mutations in regulatory genes that control embryonic development are often the cause of congenital heart defects in humans (Bruneau, 2008). Numb and Numblike are known to be involved in many processes (Gulino et al., 2010), but do these proteins also have a role in heart disease? Ultimately, understanding the regulation of the niche, and the multiplication of cardiac progenitors within it, at the molecular level may provide important clues about, and possibly treatments for, congenital heart disease.
